# An Improved Bluetooth Indoor Positioning Method Using Dynamic Fingerprint Window

**DOI:** 10.3390/s20247269

**Published:** 2020-12-18

**Authors:** Ling Ruan, Ling Zhang, Tong Zhou, Yi Long

**Affiliations:** 1 Key Laboratory of Virtual Geographic Environment, Nanjing Normal University, Ministry of Education, Nanjing 210023, China; 151301020@njnu.edu.cn (L.R.); 191302055@njnu.edu.cn (T.Z.); longyi@njnu.edu.cn (Y.L.); 2 State Key Laboratory Cultivation Base of Geographical Environment Evolution (Jiangsu Province), Nanjing 210023, China; 3 Jiangsu Center for Collaborative Innovation in Geographical Information Resource Development and Application, Nanjing 210023, China

**Keywords:** Bluetooth positioning, fingerprint window, dynamic window, positioning efficiency

## Abstract

The weighted K-nearest neighbor algorithm (WKNN) is easily implemented, and it has been widely applied. In the large-scale positioning regions, using all fingerprint data in matching calculations would lead to high computation expenses, which is not conducive to real-time positioning. Due to signal instability, irrelevant fingerprints reduce the positioning accuracy when performing the matching calculation process. Therefore, selecting the appropriate fingerprint data from the database more quickly and accurately is an urgent problem for improving WKNN. This paper proposes an improved Bluetooth indoor positioning method using a dynamic fingerprint window (DFW-WKNN). The dynamic fingerprint window is a space range for local fingerprint data searching instead of universal searching, and it can be dynamically adjusted according to the indoor pedestrian movement and always covers the maximum possible range of the next positioning. This method was tested and evaluated in two typical scenarios, comparing two existing algorithms, the traditional WKNN and the improved WKNN based on local clustering (LC-WKNN). The experimental results show that the proposed DFW-WKNN algorithm enormously improved both the positioning accuracy and positioning efficiency, significantly, when the fingerprint data increased.

## 1. Introduction

Bluetooth positioning is a hotspot in indoor positioning technology, which has the characteristics of low power consumption, easy deployment, small size, low cost, and high security [1]. Most smartphones are equipped with Bluetooth signal-receiving modules, so smartphones are typically used for indoor location-based service (LBS) applications using Bluetooth positioning in many areas, such as shopping malls, airports, and parking lots. The Bluetooth indoor positioning algorithms have been classified into three types: the proximity algorithm, the triangulation algorithm, and the scene analysis algorithm [2]. The location fingerprinting algorithm belongs to one of the scene analysis algorithms. The advantage of fingerprint positioning is that it can achieve location estimation without measuring the distance and angle between the unknown position and access points (APs), and there is no need to know the APs’ locations. Therefore, it is widely used as one of the most popular indoor positioning solutions. For fingerprint positioning based on Bluetooth, there are also two stages, the offline and online stages [3]. The offline stage involves Bluetooth device placement, the collection of the vectors of the received signal strength (RSS), and fingerprint database construction. In the online stage, the RSS vector of a new location, which needs to be measured, is obtained first. The vector is then applied to compare it with the RSS vector data in the fingerprint database to obtain its position.

From the academic perspective, fingerprint positioning algorithms can be divided into deterministic [4] and probabilistic methods [5]. Deterministic algorithms are easy to implement. They use a similarity metric to calculate the received RSS data and stored fingerprint data. Then, the positioning result is close to the fingerprint location in signal space [6]. The signal comparison method of similarity generally adopts Euclidean distance [7,8,9], cosine similarity [10], and Tanimato similarity [11,12]. The weighted K-nearest neighbor algorithm (WKNN) is an easily implemented traditional deterministic method and has been applied more widely [13]. Inertial sensors, magnetic sensors, light sensors, and other sensors are integrated into smartphones, and fusion-based indoor positioning has become more prominent in recent years [14,15]. PDR (pedestrian dead reckoning) provides the direction and distance obtained from inertial sensors [16]. PDR-based positioning methods can work well with short moving distances, and their performance may degrade when the walking distance increases [17]. Motion-assisted positioning has been fused into the triangulation or fingerprinting algorithm, achieving higher accuracy [18]. The combination of Bluetooth fingerprint positioning and inertial sensors has been shown to efficiently improve performance in many studies [19,20,21].

WKNN is widely used in indoor positioning based on Bluetooth. Although it has significant advantages compared with other algorithms, there are still some inherent problems. The first is the high computational cost in large-scale positioning regions. With the in-depth application of indoor positioning technologies in various industries, the indoor positioning scene area is getting larger, resulting in many RSS vector data in the fingerprint database. Under these circumstances, the fingerprint matching cost for WKNN is high, which is not conducive to real-time positioning. The second is the effect on the positioning accuracy of the irrelevant fingerprint data. Due to signal instability, the irrelevant fingerprint of the RSS vector is similar to the data in the fingerprint database but far away from where the actual position would exist. These fingerprint data affect and reduce the positioning accuracy when performing the matching calculation process. Therefore, selecting the appropriate similar fingerprint data from the database more quickly and accurately is an urgent problem to be solved. At present, the standard strategy for large-scale region positioning is space partitioning [22]. A large area is partitioned into small areas by location clustering methods, such as K-means clustering [23] and support vector classifier (SVC) [24]. However, the partition angle is gained from the gathered signals, which may be changing all the time. Besides, these clustering methods improve the positioning efficiency, but they ignore the irrelevant fingerprints’ interference and fail to enhance the positioning accuracy.

In this paper, inspired by window analysis and the PDR method, we realize that the range of motion space of indoor pedestrians in a certain positioning period is relatively limited and predictable when the current position, speed, and direction of the indoor environment are known. Only the fingerprint data in the above range are required to perform the matching calculation. Based on the above analysis, this paper proposes a dynamic fingerprint window (DFW) to improve the Bluetooth indoor positioning method based on a weighted k-nearest neighbor (WKNN). The dynamic fingerprint window is a spatial range for local searching from fingerprint data, and it can be dynamically adjusted according to the indoor pedestrian movement. The window always covers the maximum possible range of the next position. The improved positioning method’s implementation via a dynamic fingerprint window is presented, which is called DFW-WKNN. Unlike the perspective on Bluetooth signal distribution characteristics in the existing literature, this article starts from the indoor pedestrian movement space to improve positioning efficiency. Finally, the method was tested and evaluated in two real typical scenarios, comparing two existing algorithms, he traditional WKNN and the improved WKNN based on local clustering (LC-WKNN). The experimental results show that the proposed DFW-WKNN algorithm enormously improved both the positioning accuracy and positioning efficiency, significantly, when the fingerprint data wee increased.

The remainder of this paper is structured as follows. Section 2 introduces the related work on the WKNN and its optimization method. Section 3 presents the specific method for the DFW-WKNN and the dynamic fingerprint window generation in detail. In Section 4, the testing of the proposed DFW-WKNN method in two experiments is described, and the results are provided, comparing them with those of the traditional WKNN and LC-WKNN positioning algorithms. Section 5 is the conclusions and future research.

## 2. Related Work

Fingerprint positioning is the most commonly used positioning method for Bluetooth indoor positioning technology. The K-nearest neighbors (KNN) algorithm is the most commonly used deterministic localization algorithm in fingerprint localization. The KNN algorithm is simple and easy to implement. After receiving the RSS vector, the KNN algorithm searches the fingerprint database and finds the K fingerprint data similar to the RSS fingerprints. Then, calculating the center of the sampling point corresponding to the K fingerprints provides a positioning result. The method is fast and straightforward, but it does not consider that the K similar fingerprints have different positioning contributions. Based on the original KNN algorithm, WKNN algorithms [2] were proposed, weighting the nearest neighboring fingerprints.

In the past decade, improvement based on the WKNN algorithm has been a research focus. The research in this field can be summarized into two aspects. The first is optimizing the computation model in the fingerprint matching, and it includes AP selection, weight sets, and similarity calculation adjustment. The IWKNN positioning method combines Isomap and WKNN for Bluetooth positioning, and the distance of different RSSI vectors is measured by the Euclidean distance of low-dimensional embeddings [25]. Adjusting the weight of adjacent reference nodes based on WKNN can improve performance by increasing the accuracy by 33.82% [26]. In the different steps of the position estimation procedure, various metrics are adopted to establish a mixed approach [27]. The positioning accuracy of the WKNN fingerprint localization algorithm is under the influence of K value change [28]. The experimental results show that the mean positioning error in KNN and Euclidean distance can reach the smallest values when *K* = 4 [29].

The second aspect of the improvement is selecting the relevant fingerprint data in the matching process. By providing a higher degree of similarity in fingerprints, the goal of improving the positioning accuracy is achieved. The method for fingerprint data storage and retrieval is important for positioning efficiency. Compared with the traditional linear scanning method, the *k*-*d* tree and the best bin first (BBF) algorithm can reduce computational costs [30]. In a real-time fingerprint-based wireless positioning system, Atia et al. proposed a faster feature reduction approach under a fast orthogonal search, selecting the most informative features. The method outperforms principal component analysis (PCA) in terms of speed [31]. Reducing the number of data in fingerprint matching is necessary for large-scale positioning regions. The common solution is to split the positioning area into sub-regions by location clustering [32]. A comparison of the fingerprint clustering algorithms is shown in Table 1, and the performance improvement is compared to the standard WKNN algorithm. The classical *K*-means [23] clustering algorithm can divide the fingerprints, but the result is influenced by the initial K-value selection and the initial cluster center. The fuzzy C-means (FCM) [33] clustering algorithm solves the problem that the location fingerprint belongs to only one fingerprint class when the pedestrian’s location is in the transition region of several fingerprint classes. Affinity propagation clustering can generate the number of classifications and the center quickly and automatically, but the accuracy improvement is limited [34].

In general, previous studies have conducted much work to optimize the fingerprint matching process, and many improvement algorithms based on WKNN have been put forward. However, most current research on the fingerprint matching process mainly analyzes the fingerprint dataset’s clustering and partitioning characteristics. They are based on the distribution and propagation of the signals. The unstable signal changes may affect the final positioning accuracy. The existing research does not pay enough attention to the improvement of positioning efficiency. Besides, indoor pedestrian activity is not considered, and the indoor space structure constraints are not enough. Therefore, this paper starts from indoor pedestrians’ activity space and proposes a dynamic fingerprint window to reduce the fingerprint data in the fingerprint match calculation. In the window, the irrelevant fingerprints are excluded to improve the positioning accuracy.

## 3. Methods

### 3.1. Overview

**Figure 1:**
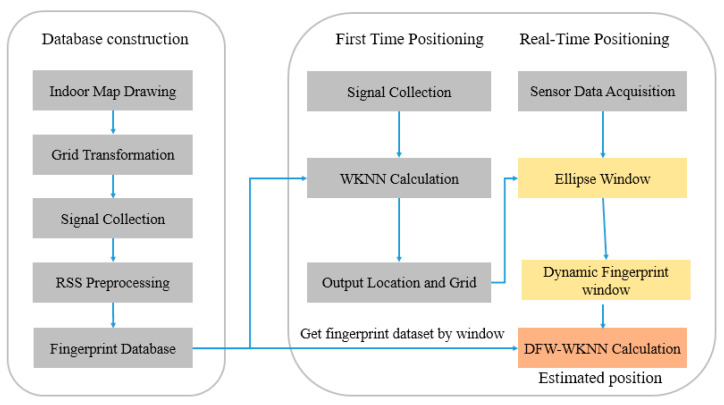
The overall structure of the dynamic fingerprint window–weighted K-nearest neighbor algorithm (DFW-WKNN) indoor positioning method.

Starting with how to select some suitable fingerprint data from the database to perform the matching calculation, this paper proposes an improved Bluetooth indoor positioning method using a dynamic fingerprint window. The optimization method is implemented based on the traditional WKNN positioning algorithm. The so-called dynamic fingerprint window is a search window used to reduce the fingerprint matching range and improve the positioning accuracy and efficiency. The range of dynamic fingerprint windows can be dynamically calculated according to the pedestrian’s real-time motion state, which can always cover the next movement of the pedestrian. The overall architecture of the proposed method is shown in Figure 1. 

The proposed method consists of the following three phases.

**Phase1**: Fingerprint database construction. This is the offline stage. It involves indoor map drawing, grid transformation, Bluetooth signal collection and preprocessing, and data storage. In this phase, the eight-direction index must be created when collecting the Bluetooth signal in each reference point (detailed in Section 3.2).

**Phase2**: First-time positioning. After collecting the current position’s Bluetooth signal, the initial location is estimated based on the WKNN fingerprint positioning algorithm. Simultaneously, the location and the Grid ID where the result is located should be recorded (detailed in Section 3.4).

**Phase3**: Real-time positioning. According to the sensor data received by the smartphone, combined with the last positioning result and Grid ID, the pedestrian dead reckoning (PDR) algorithm is used to calculate the parameters of the ellipse window. The dynamic fingerprint window range is calculated when combined with the indoor grid (detailed in Section 3.3). The fingerprint dataset is selected quickly using the eight-direction index in the database. The final result is estimated using the fingerprint data in the dynamic fingerprint window and the WKNN positioning algorithm (detailed in Section 3.4).

### 3.2. Fingerprint Database and Eight-Direction Index

Most of the localization methods based on RSS share the same fundamentals. The indoor map drawing and Bluetooth beacon placement must be completed first to build the indoor localization environment. Then, the fingerprint database can be created, and the RSS distributions of all the Bluetooth beacons need to be stored at all the reference points (RPs). In the indoor map, space is usually divided into 1 m * 1 m grids [38], and the center point of each grid is regarded as the reference point. The RSS value has a strong fluctuation due to the multipath propagation of signals reflected in walls, rooms, and floors. Therefore, in fingerprint data collection, the weakest RSS observations of a reference point need to be abandoned, and the remainder of RSS observations should take the average as the RSS measurement to reduce these influences. In traditional fingerprint database construction, the expected format is as shown in Table 2. The coordinates of each reference point and the corresponding RSS collected are stored.

**Figure 2:**
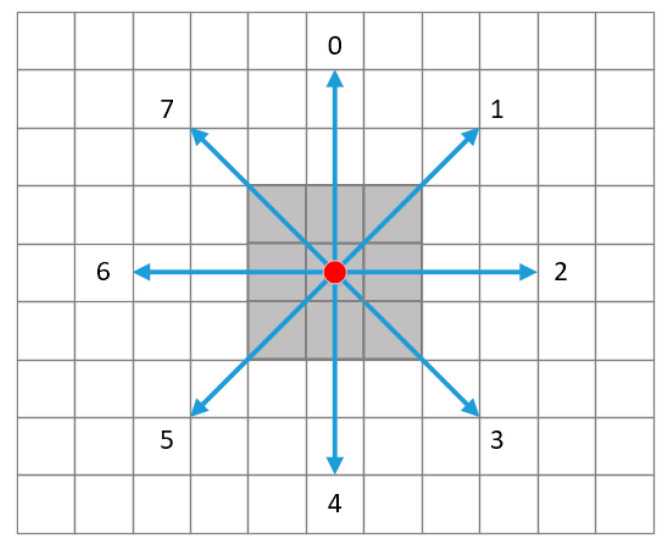
The eight-direction index of the fingerprint database.

In the process of fingerprint data matching, the computation is costly when the data of every reference point in the database participate in the matching calculation. Large-scale positioning regions must localize the positioning model into a sub-region by location clustering [20]. K-means clustering and support vector classifier methods are used in much research to reduce the computation costs. In this paper, an efficient method for fingerprint data retrieval is proposed, based on spatial proximity. An eight-direction index is constructed during the fingerprint database construction to build the next dynamic fingerprint window to find suitable reference points quickly. The eight-direction index of the fingerprint database is shown in Figure 2. When collecting the Bluetooth signal of a reference point, the reference points in the surrounding eight directions need to be collected, and the relationship needs to be recorded.

The storage format of the fingerprint database based on the eight-direction index is shown in Table 3. Compared with the traditional fingerprint database structure, the NearRP field is added, and the adjacent reference points are stored. The value of the direction_id ranges from 0 to 7. Thereby, all the reference points are connected through the eight-direction index, convenient for searching the required fingerprint data quickly in real-time positioning.

### 3.3. Dynamic Fingerprint Window

In the traditional fingerprint positioning method, the RSS value received in real time is generally matched with each reference point’s RSS collection in the fingerprint database. However, this method requires many matching calculations, and irrelevant fingerprints interfere with the location estimation. In fact, only the fingerprint data within the maximum range of the user’s movement in the positioning period are needed to perform the matching calculation. The maximum range of the user’s movement is the proposed dynamic fingerprint window in this article, and it can always cover the space that the user can reach during a positioning period. Compared with the fingerprint clustering range proposed from the perspective of signal distribution in the related literature, the dynamic fingerprint window starts from indoor pedestrian movement, which is more scientific and reasonable.

**Figure 3:**
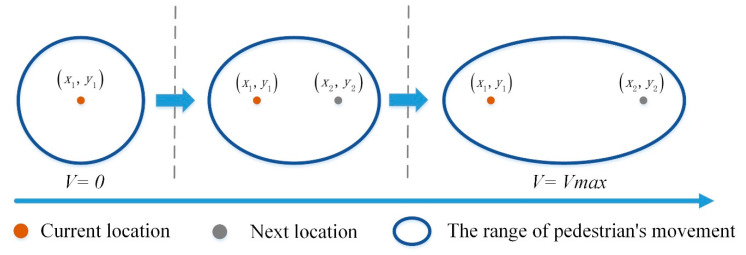
Pedestrian motion range and elliptical window.

Pedestrians are in different motion states in indoor positioning, such as stationary, slow walking, fast walking, and running. The range and form of the dynamic fingerprint window should be different, as shown in Figure 3. In an ideal barrier-free indoor space, the range of pedestrian movement in a positioning period should be a standard circle when the pedestrian is at a standstill, and movement may occur in all directions. When the pedestrian’s speed is not 0, the range of movement can be an ellipse, and the direction of the semi-major axis in the ellipse is consistent with the direction of the pedestrian movement. As the speed increases, the short half axis of the ellipse becomes smaller, the semi-major axis becomes more prolonged, and the ellipse stretches in the direction of the pedestrian movement.

**Figure 4:**
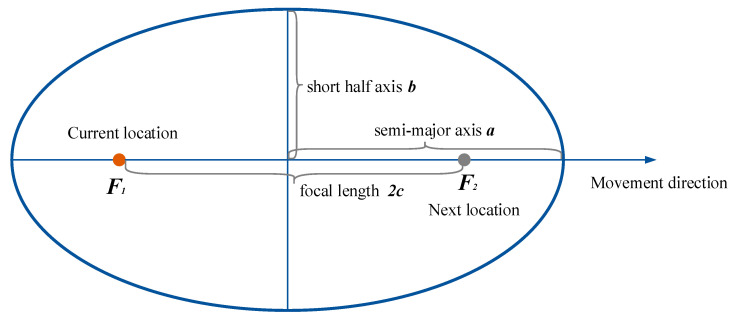
Conceptual model of the ellipse window.

According to the above analysis, the range of pedestrian movement that is the dynamic fingerprint window can be described as an ellipse window, and the conceptual model is shown in Figure 4. The two focal points of the ellipse are composed of the current position $F_{1}\left(x_{1},y_{1}\right)$ and the next predicted position $F_{2}\left(x_{2},y_{2}\right)$. The X-axis is established based on the straight line of $F_{1}F_{2}$, and the Y-axis is established based on the vertical line of $F_{1}F_{2}$. The direction of the semi-major axis of the ellipse is the same as the predicted movement direction of the pedestrian. The range formed by the ellipse is the area in which pedestrians may appear in a positioning cycle. The next predicted position can be estimated from the sensor data recorded by the smartphone.

In order to determine the range of the ellipse window, the first step is to estimate the next predicted position and focal length of the ellipse. Although the predicted position needs to be calculated here, this is only a rough positioning estimation and cannot replace the final position calculation. According to the PDR algorithm, the acceleration sensor and orientation sensor data are collected by the inertial measurement unit in the smartphone, and the calculation of the next position and focal length L is based on the following formula:

(1) $$x_{2}=x_{1}+\sum_{n=1}^{k}d_{n}sin\theta_{n},$$

(2) $$y_{2}=y_{1}+\sum_{n=1}^{k}d_{n}cos\theta_{n},$$

(3) $$L=\sqrt{{\left(x_{2}-x_{1}\right)}^{2}+{\left(y_{2}-y_{1}\right)}^{2}},$$

where $\left(x_{2},y_{2}\right)$ indicates the coordinates of the next position, $\left(x_{2},y_{2}\right)$ are the known coordinates of the previous position, *d* is the step length, *k* is the step count, and θ represents the heading angle. The step size estimation is not the focus of this article, and a fixed step size is generally used. After the focal length of the ellipse window is obtained, the parameter equation of the ellipse can be determined by calculating the short axis length *b*. 

As we know, the length of the short half axis in the ellipse window is related to the pedestrian’s speed. The greater the speed, the smaller the short half axis. When the pedestrian is stationary, the lengths of the semi-major axis and short half axis in the ellipse are equal, and the ellipse window becomes a standard circle; its radius should cover the range where the pedestrians appear farthest. At this time, the calculation formula for the length in the short half axis is as follows:

(4) $$b=V_{max}\;*\;T+error,$$

where $V_{max}$ represents the maximum speed value of the pedestrian in the indoor environment. According to Chandra’s research [39], indoor pedestrians’ maximum walking speeds do not exceed 3 m/s. Therefore, $V_{max}$ is set to 3 m/s in this paper, *T* is the positioning period, and *error* is the general empirical error of Bluetooth fingerprint positioning, which is set to 3 m.

When the speed of pedestrians increases and exceeds $V_{max}$, the length of the short half axis in the ellipse window would become very small, but there must be a minimum value. The general empirical error of Bluetooth fingerprint positioning can be used as the minimum length of the short half axis in the ellipse window, and the length of the short half axis should be within the interval of $\left[error,V_{max}\;*\;T+error\right]$.

When the speed of pedestrians *V* is within $\left[0,V_{max}\right]$, the length of the short half axis in the ellipse window is inversely proportional to the speed. At this time, the value of *b* is calculated as follows:

(5) $$b=\left(V_{max}-V\right)*T+error.$$

Among them, the speed can be calculated by L/T. Therefore, when the previous position is known, the PDR algorithm can be used to estimate the parameters *c* and *a* in the ellipse equation, and the range of the ellipse window can be finally determined.

**Figure 5:**
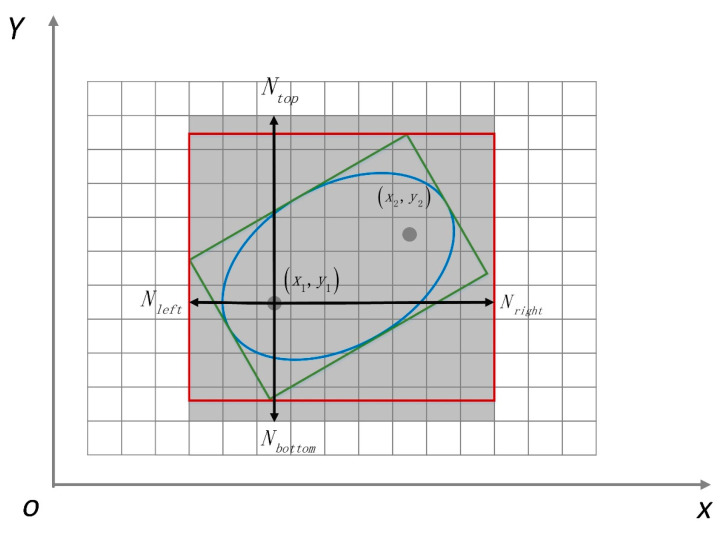
The shape conversion of the dynamic fingerprint window. The ellipse window is converted to the secondary circumscribed rectangle.

However, the ellipse window is composed of arcs, and its range is irregular, which is not conducive to quickly determining which grids are within the ellipse window unless every reference point is involved in the calculation of the ellipse equation. Therefore, after calculating the ellipse window’s parameters, the window needs to be combined with the indoor spatial grid to simplify the search algorithm to find the fingerprint data to be matched as soon as possible. This paper proposes converting the ellipse window into a rectangular window parallel to the grid direction so that the space range of the required fingerprint data can be quickly determined in the real-time positioning process. The shape conversion of the dynamic fingerprint window is shown in Figure 5. The circumscribed rectangle of the ellipse window is firstly generated, but the circumscribed rectangle is not parallel to the coordinate axis. It is inconvenient to quickly obtain fingerprint data during retrieval, so the secondary circumscribed rectangle is generated based on the original circumscribed rectangle. The RSS collection in the reference points within the secondary circumscribed rectangle is the fingerprint dataset that is needed to perform the matching calculation. 

Assuming that the current position of the pedestrian is $F_{1}$ and the coordinates are $\left(x_{1},y_{1}\right)$, the length of the semi-major axis *a*, the length of the short half axis *b*, and the focal length *2c* can be quickly calculated based on the above calculation method for the ellipse window. At this moment, the current position of the pedestrian is regarded as the center point; the range of the fingerprint dataset that is needed to perform the matching calculation is in the following:

(6) $$N_{left}=\lceil \frac{\left(a-c\right)\cos\alpha +b\sin\alpha }{cellsize}\rceil ,$$

(7) $$N_{right}=\lceil \frac{\left(a+c\right)\cos\alpha +b\sin\alpha }{cellsize}\rceil ,$$

(8) $$N_{bottom}=\lceil \frac{\left(a-c\right)\sin\alpha +b\cos\alpha }{cellsize}\rceil ,$$

(9) $$N_{top}=\lceil \frac{\left(a+c\right)\sin\alpha +b\cos\alpha }{cellsize}\rceil ,$$

where $N_{left}$, $N_{right}$, $N_{bottom}$, and $\;N_{top}$ are the numbers of grids that need to be displaced to the left, right, down, and up above the current position. The cell size represents the grid size of the indoor positioning environment, and α is the angle between the semi-major axis of the ellipse and the horizontal grid, which is calculated from the point $F_{1}$ and point $F_{2}$. Under the condition that the number of grid points $F_{1}$ is known, the fingerprint dataset covered by the dynamic fingerprint window that is the displacement range in the four directions can be quickly selected from the database through the established eight-direction index. The pseudocode of Algorithm 1 that represents the calculation process for a dynamic fingerprint window is listed below.
**Algorithm 1:** the proposed dynamic fingerprint window calculation algorithm**Input**: current location $F_{1}$$\left(x_{1},y_{1}\right)$, positioning cycle *T*, and cell size cz;step count *N* and heading angle $\left(\theta_{1},\;\theta_{2},\ldots ,\theta_{i},\ldots ,\theta_{n}\right)$;Define *error* = 3, $V_{max}$ = 3;**1.**  calculate the next predicted position $F_{2}$$\left(x_{2},y_{2}\right)$ and focal length *L*;**2.**  **c** = *L/2*;**3.**  *V* = *L/T*;**4.**  **if***V != 0***5.**   $b=V_{max}*T+error$;**6.**  **else****7.**   $b=\left(V_{max}-V\right)*T+error$;**8.**  $a=\;\sqrt{b^{2}+c^{2}}$;**9.** /* The parameters of the ellipse equation have been calculated*/ **10.**  convert the ellipse window to the secondary circumscribed rectangle;**11.** calculate the displacement range [$N_{left}$, $N_{right}$,$\;N_{bottom}$,$\;N_{top}$];**Output**: the displacement range [$N_{left}$, $N_{right}$,$\;N_{bottom}$,$\;N_{top}$];

### 3.4. DFW-WKNN Positioning Method

**Figure 6:**
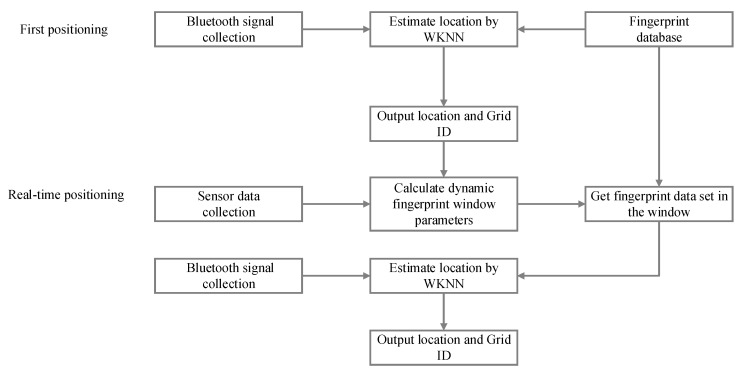
The flow chart of the DFW-WKNN indoor positioning method.

The indoor positioning algorithm based on the dynamic fingerprint window includes two parts: the first-time positioning and real-time positioning. The flow chart of the algorithm is shown in Figure 6. After the fingerprint database construction, the traditional WKNN positioning method is used for the first-time positioning to obtain the output location and Grid ID. Then, the inertial sensors’ data are collected, and the dynamic fingerprint window is calculated when the data are combined with the former location. Finally, the accurate location is estimated by selecting the appropriate fingerprint dataset under the constraint of the dynamic fingerprint window.

In the first-time positioning stage, the grid number where the positioning result is located is recorded as a priori knowledge and provided for real-time positioning to improve efficiency. Since the current location must be known before calculating the range of the dynamic fingerprint windows, the initial position must be determined first. The traditional WKNN positioning result, outdoor GPS position, or input determined position can be used to achieve the initial position. The higher the accuracy of the first positioning, the better the result in real-time positioning.

In the real-time positioning stage, the dynamic fingerprint window range can be calculated when firstly combining the grid number in the first positioning result. Then, the corresponding fingerprint dataset in the database can be selected quickly based on the eight-direction index. Finally, the obtained RSS collection from an unknown location matches the selected fingerprint dataset to realize real-time position estimation. The specific steps in the real-time positioning stage are as follows:

**Step1**: According to the accelerometer data received by the inertial measurement unit of the smartphone, the number of steps $N_{step}$ within the positioning period *T* is estimated based on the wave peak detection algorithm.

**Step2**: Set a fixed experience value as the average step length and combine it with the number of steps and the last positioning result $P_{1}\left(x_{1},y_{1}\right)$ to estimate the parameters a, b, and c of the ellipse window.

**Step3**: Calculate the range of the dynamic fingerprint window $W_{o}$ based on the ellipse window parameters and grid size, which contains the number of grids that need to be displaced from the up, down, left, and right directions at the current position. 

**Step4**: With the support of the eight-direction index, select the fingerprint dataset $F=\left\{F_{1},F_{2},\ldots ,F_{i},\ldots ,F_{n}\right\}$ from the fingerprint database based on the range of the dynamic fingerprint window $W_{o}$.

**Step5**: Obtain the signal data $RSS_{p}=\left(rss_{p1},rss_{p2},\ldots ,rss_{pn}\right)$ at the current position, match the signal data with the selected fingerprint dataset F one by one, and calculate the similarity between $RSSI_{p}$ and $F_{i}$ using the Euclidean distance, using the following formula:

(10) $$D_{i}=\sqrt{\sum_{j=1}^{n}{\left(RSS_{j}-RSS_{ij}\right)}^{2}}.$$

**Step6**: Sort the calculated $D_{i}$ by descending, select the top K grids with the smallest similarity between their fingerprint data and the current signal data, and calculate the weight of each grid, using the following formula:

(11) $$W_{i}=\frac{d_{i}}{\sum_{j=1}^{K}d_{i}}.$$

**Step7**: The coordinates corresponding to the K grids are weighted and averaged as the final positioning coordinates, and the coordinates $\hat{P}\left(x,y\right)$ and the number of grids that $\hat{P}\left(x,y\right)$ located are output. The positioning calculation uses the following formula:

(12) $$\hat{P}\left(x,y\right)=\sum_{i=1}^{K}W_{i}Pos_{i}\;,$$

where $Pos_{i}$ represents the coordinates of the selected top *K* grids.

## 4. Experimental Results

### 4.1. Experimental Scenarios and Data Preparation

**Figure 7:**
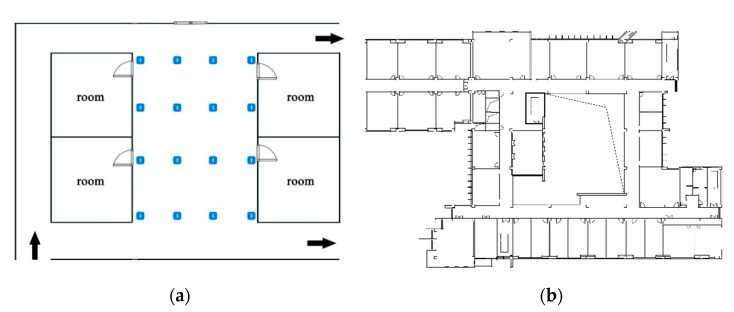
Indoor positioning experimental scenarios. (**a**) A rectangular indoor positioning experimental scenario on the third floor of an office building, and (**b**) a relatively complex indoor experimental scenario on the fourth floor of the School of Geographical Sciences at Nanjing Normal University.

To verify the proposed DFW-WKNN algorithm’s performance, we purchased some mature Beacon facilities from a commercial company to implement an indoor positioning prototype system based on DFW-WKNN, which is run on Android smartphones. The traditional WKNN positioning algorithm and the improved WKNN positioning algorithm based on K-means location clustering (LC-WKNN) were also integrated into the prototype system. LC-WKNN is a common solution for large-scale positioning regions in the current research literature. According to previous research, the empirical K’s value in the three algorithms is always set to 4. The positioning accuracy and positioning efficiency of the above three algorithms were compared through the experimental results. Two real experimental scenarios were set up. The layout of the scenarios is shown in Figure 7. Experimental Scenario 1 is relatively small, and it is a partial area of the third floor of an office building in an industrial park in Nanjing, China. Experimental Scenario 2 is a relatively large area on the fourth floor of the School of Geographical Sciences at Nanjing Normal University, China. Besides, the shapes of the two experimental scenarios are different. Scenario 1 is a regular rectangle, and the positioning area in Scenario 2 is a homocentric square corridor. The iBeacon deployment spacing of the two scenarios is 3 and 6 m, respectively, and the detailed parameters of the two experimental scenarios are shown in Table 4.

In the fingerprint database construction, more than ten samples of RSS data were collected at the center point in each grid. The smartphone used in the real experimental scenarios was a HUAWEI Mate 30 Pro. The update rates of the accelerometer and gyroscope in the smartphone were set to 100 Hz, and the update interval of the iBeacon receiver was set to 1 s. During the experiment, a tester held the mobile phone with the developed prototype system and walked freely with a non-uniform speed in two experimental scenarios. We selected 35 points and 70 points as the test points in Experimental Scenarios 1 and 2, respectively. Five sets of positioning results were collected in each experimental scenario. The positioning results were recorded under the above three algorithms, which have no other constraints.

### 4.2. Results for Positioning Accuracy

**Figure 8:**
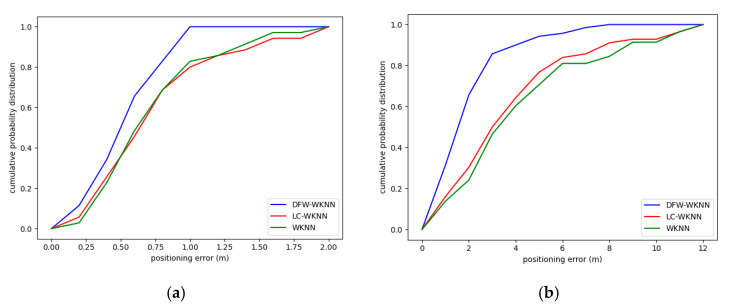
The cumulative probability distributions of the positioning errors of the WKNN, local clustering (LC)-WKNN, and DFW-WKNN positioning algorithms in two experimental scenarios. (**a**) The results in Experimental Scenario 1; (**b**) The result in Experimental Scenario 2.

The cumulative probability distributions of the positioning errors of the WKNN, LC-WKNN, and DFW-WKNN positioning algorithms in the two experimental scenarios are shown in Figure 8. The positioning error statistics for the above three positioning algorithms are listed in Table 5. The results show that the DFW-WKNN positioning algorithm is significantly better than the LC-WKNN algorithms. Through the constraints of the dynamic fingerprint window, the positioning accuracy of traditional WKNN is much improved.

The average error of the DFW-WKNN positioning algorithm was 0.51 m in Experimental Scenario 1, where the beacon deployment spacing was 3 m; its improvement was 27.14% and 28.17%, and the RMSE (Root Mean Squared Error) improvement was 28.75% and 31.32%, when compared with the traditional WKNN and the improved LC-WKNN positioning algorithms. The average error for DFW-WKNN was 1.86 m in Experimental Scenario 2, where the beacon deployment spacing was 6 m; its improvement was 53.96% and 50.13%, and the RMSE improvement was 52.48% and 48.93%. Therefore, the experimental results from the two different scenarios prove that the proposed DFW-WKNN can improve the WKNN and LC-WKNN algorithms’ positioning accuracy.

**Figure 9:**
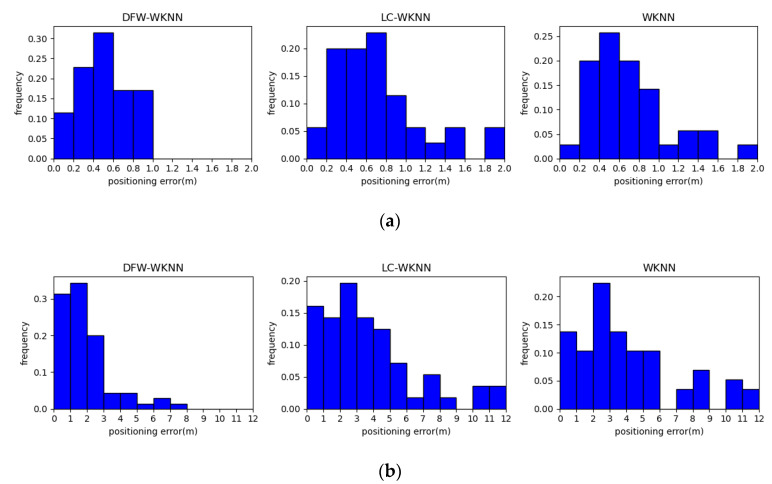
The positioning error distributions for the WKNN, LC-WKNN, and DFW-WKNN positioning algorithms. (**a**) Comparison of the positioning error distributions in Experimental Scenario 1; (**b**) Comparison of the positioning error distributions in Experimental Scenario 2.

Additionally, the maximum positioning error can be effectively controlled through the constraints of the dynamic fingerprint window. The distribution positioning errors of the three algorithms in two experimental scenarios are shown in Figure 9. The results for the WKNN and LC-WKNN algorithms are relatively scattered, and their quartile deviations are 3.40 and 3.07, respectively. The positioning error distribution for the proposed algorithm is more concentrated than the other two, and its quartile deviation is 1.72. 

**Figure 10:**
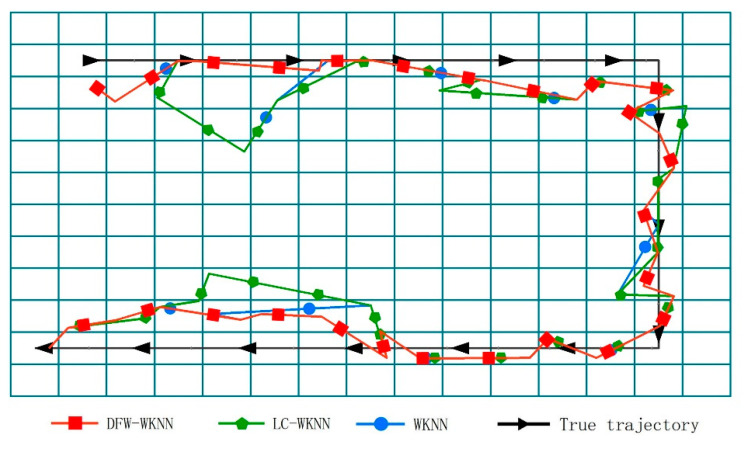
Comparing the trajectories for WKNN, LC-WKNN, DFW-WKNN, and a real pedestrian in Experimental Scenario 1.

**Figure 11:**
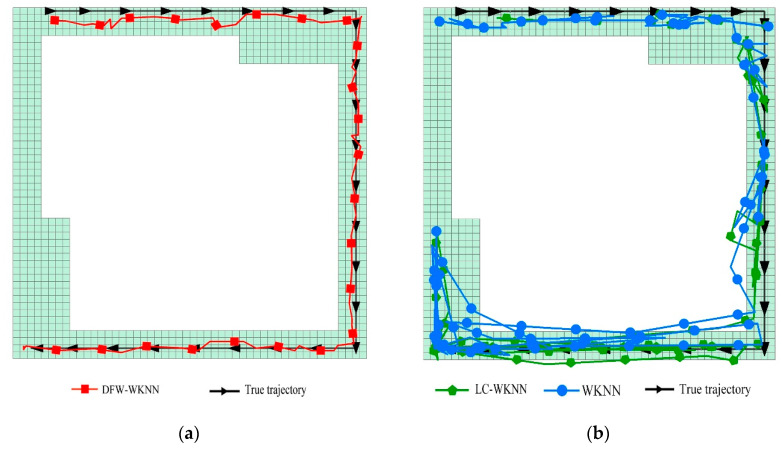
Comparing the trajectories for WKNN, LC-WKNN, DFW-WKNN, and a real pedestrian in Experimental Scenario 2. (**a**) The trajectories for DFW-WKNN and a real pedestrian; (**b**) The trajectories for WKNN, LC-WKNN, and a real pedestrian.

The positioning trajectories of WKNN, LC-WKNN, and DFW-WKNN in the two experimental scenarios are visualized, shown in Figure 10 and Figure 11. Due to the instability of the received Bluetooth signal, the trajectories of WKNN and LC-WKNN have prominent jump phenomena in the forward direction, especially in Experimental Scenario 2, where the beacon deployment spacing was 6 m. When the pedestrians were stationary, the trajectories swung around the real position. Because the dynamic fingerprint window can limit the offset of each positioning result, the positioning trajectory of DFW-WKNN is very similar to the actual motion trajectory.

### 4.3. Results for Positioning Efficiency

The positioning efficiency depends on the computational complexity of the positioning algorithms. Among the three algorithms implemented in the prototype system, no matter how large the number of fingerprint datasets, the calculation amount of the DFW-WKNN positioning algorithm is relatively fixed, and its time complexity is O(1). The time complexity of the traditional WKNN positioning algorithm is $O\left(n^{2}\right)$; its positioning time significantly rises as the number of fingerprints increases. The time complexity of the LC-WKNN positioning algorithm is $O\left[{\left(n/k\right)}^{2}\right]$, and *k* is the number of fingerprint clusters, which determines the positioning time.

The calculation time for location estimation in the real-time positioning stage was recorded in the developed prototype system. The positioning time statistics for WKNN, LC-WKNN, and DFW-WKNN are listed in Table 6. The average positioning time for DFW-WKNN was 4.37 ms, and the average time reductions in Scenarios 1 and 2 were 23.07 and 89.53 ms compared with the WKNN algorithm. The improvement effect is pronounced. Compared with the LC-WKNN, which is considered the commonly used method for large-scale positioning regions, the average time reductions in Scenarios 1 and 2 were 1.32 and 13.05 ms, respectively. The DFW-WKNN algorithm still shows a certain improvement in positioning efficiency.

**Figure 12:**
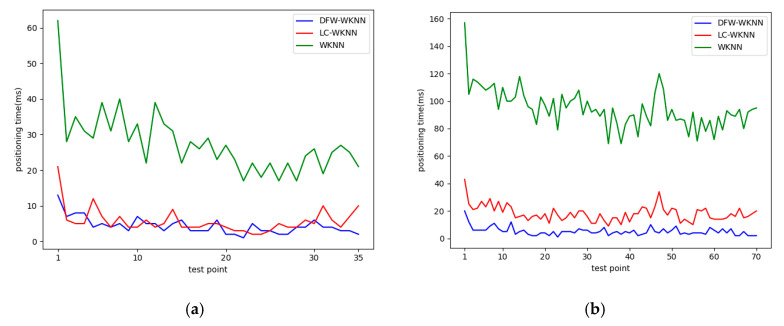
The positioning time of each test point for WKNN, LC-WKNN, and DFW-WKNN. (**a**) The positioning time of each test point in Experimental Scenario 1; (**b**) The positioning time of each test point in Experimental Scenario 2.

The positioning time of each point in the three algorithms is shown in Figure 12. In the two experimental scenarios, the LC-WKNN algorithm still greatly improved the WKNN algorithm’s positioning efficiency. In Experimental Scenario 1 with 180 fingerprint data, the positioning time difference between LC-WKNN and DFW-WKNN was not considerable. Due to the small number of fingerprint data in a database, there is little difference between the number of fingerprint data in a location clustering and dynamic fingerprint window, and the positioning times in LC-WKNN and DFW-WKNN showed similar performance in Scenario 1. In Experimental Scenario 2, with 868 fingerprint data, the average reduction in positioning time was 13.05 ms, and the optimization effect was prominent when the number of fingerprints in the database increased.

### 4.4. Conclusions

In this study, a developed prototype system collected data in two real experimental scenarios. The results for the traditional WKNN, LC-WKNN, and DFW-WKNN algorithms are compared in terms of positioning accuracy and efficiency. Through the above analysis, we know that:

(1) The proposed DFW-WKNN algorithm significantly improves the traditional WKNN algorithm in terms of positioning accuracy. The average error improvements were 27.14% and 53.96% in the above two typical experimental scenarios. The maximum positioning error can be effectively controlled, and the error distribution is more concentrated.

(2) The proposed DFW-WKNN algorithm greatly improves the traditional WKNN algorithm in terms of positioning efficiency. The average positioning time improvements were 84.08% and 94.57% in the above two typical experimental scenarios. The efficiency improvement becomes more apparent when the number of fingerprints in the database increases.

(3) The proposed DFW-WKNN outperforms the LC-WKNN positioning algorithm in terms of both positioning accuracy and efficiency. The average error improvements were 28.17% and 50.13%, and the average positioning time improvements were 23.19% and 71.74%, in the above two typical experimental scenarios. The efficiency improvement was more prominent in large-scale positioning regions.

The proposed dynamic fingerprint window improves the traditional WKNN positioning algorithm by selecting the fingerprint data in the indoor pedestrian movement range, which solves the interference from some irrelevant fingerprints far away from the actual position. Compared with the WKNN improvement algorithm based on fingerprint clustering, the optimization effect of DFW-WKNN is noticeable. When compared with some complex fusion models such as Kalman filtering and hidden Markov models, the improvement effect of the proposed method is not prominent. Still, the DFW-WKNN algorithm’s complexity is very low and easy to understand, which is a great feature and advantage. In terms of positioning efficiency, the DFW-WKNN algorithm’s optimization effect is very prominent, especially for large-scale positioning scenes. Unlike the common fingerprint clustering algorithm, the proposed algorithm takes the former location as the prior knowledge, and the computational cost is relatively fixed.

## 5. Summary and Future Work

The traditional WKNN algorithm in large-scale positioning regions would lead to high computation expenses, which would affect positioning efficiency. Due to signal instability, some fingerprints reduce positioning accuracy when performing the matching calculation process. Aiming to select appropriate similar fingerprint data from the database more quickly and accurately, this paper presents an improved Bluetooth indoor positioning method using the dynamic fingerprint window for the above problem. The dynamic fingerprint window is used to reduce the searching range for fingerprint data, and it can be calculated by the indoor pedestrian movement. Compared with the traditional WKNN algorithm, the proposed DFW-WKNN positioning algorithm dramatically improved the positioning accuracy and efficiency. The comparison results also demonstrate that the proposed DFW-WKNN outperforms the LC-WKNN positioning algorithm, considering the commonly used methods for large-scale positioning regions. The efficiency improvement became more apparent when the number of fingerprints in the database increased, and the positioning accuracy was greatly improved. Our future research will apply the proposed DFW-WKNN positioning method to other positioning technologies, such as Wi-Fi positioning, LED visible light positioning, deep learning algorithms, and multi-technology integration positioning.

## Figures and Tables

**Table 1:** Comparison of the fingerprint clustering algorithms.

Method	Performance	Limitation
K-means clustering [34]	The accuracy improvement is 21.1%	The number of clusters needs to be determined
Fuzzy C-means clustering [35]	The accuracy is enhanced slightly; efficiency improvement is 66%.	The accuracy improvement is not obvious
Affinity propagation clustering [34]	The accuracy improvement is 7.28%	The complexity is high when the amount is large
Adaptive hierarchical clustering [36]	The accuracy improvement is 17.6%	The efficiency optimization is not reflected
Smallest enclosing Circle clustering [37]	The accuracy improvement is 11.9%	The implementation depends on unstable path-loss model

**Table 2:** The format of the traditional fingerprint database.

RPs	X	Y	$AP_{1}$	$AP_{2}$	$AP_{3}$	…	$AP_{M}$
1	$x_{1}$	$x_{1}$	$RSS_{1}^{1}$	$RSS_{1}^{2}$	$RSS_{1}^{3}$	…	$RSS_{1}^{M}$
2	$x_{2}$	$x_{2}$	$RSS_{2}^{1}$	$RSS_{2}^{2}$	$RSS_{2}^{3}$	…	$RSS_{2}^{M}$
…	…	…	…	…	…	…	…
N	$x_{N}$	$x_{N}$	$RSS_{N}^{1}$	$RSS_{N}^{2}$	$RSS_{N}^{3}$	…	$RSS_{N}^{M}$

**Table 3:** The storage format of the fingerprint database based on the eight-direction index.

RPs	Coordinate	Time	NearRP	APs	RSS
1	($x_{1},y_{1}$)	$t_{1}$	($\mathrm{driction}\_{\mathrm{Id}}_{1}$, $\mathrm{rp}\_{\mathrm{id}}_{1}$)	{$AP_{1}^{1}$, $AP_{1}^{2},\ldots$}	{$RSS_{1}^{1}$, $RSS_{1}^{2},\;\ldots$}
2	($x_{2},y_{2}$)	$t_{2}$	($\mathrm{driction}\_{\mathrm{Id}}_{2}$, $\mathrm{rp}\_{\mathrm{id}}_{2}$)	{$AP_{2}^{1}$, $AP_{2}^{2},\ldots$}	{$RSS_{2}^{1}$, $RSS_{2}^{2},\;\ldots$}
…	…	…	…	…	…
*N*	($x_{N},y_{N}$)	$t_{N}$	($\mathrm{driction}\_{\mathrm{Id}}_{N}$, $\mathrm{rp}\_{\mathrm{id}}_{N}$)	{$AP_{N}^{1}$, $AP_{N}^{2},\ldots$}	{$RSS_{N}^{1}$, $RSS_{N}^{2},\;\ldots$}

**Table 4:** The conditions of two experimental scenarios.

Scenarios	Area	Grid Size	iBeacon Spacing	Quantity of APs	Quantity of RPs
1	120 m^2^	0.6 m × 0.9 m	3m	16	180
2	300 m^2^	0.6 m × 0.6 m	6m	36	868

**Table 5:** The positioning error statistics for the WKNN, LC-WKNN, and DFW-WKNN.

Scenarios	Method	Average Error	Max. Error	RMSE	95% Confidence Interval
Experimental Scenario 1 (*m*)	WKNN	0.70	1.80	0.80	(0.57, 0.83)
	LC-WKNN	0.71	1.86	0.83	(0.56, 0.86)
	DFW-WKNN	0.51	0.89	0.57	(0.43, 0.59)
Experimental Scenario 2 (*m*)	WKNN	4.04	11.87	5.03	(3.27, 4.82)
	LC-WKNN	3.73	11.40	4.68	(2.98, 4.47)
	DFW-WKNN	1.86	7.6	2.39	(1.51, 2.22)

**Table 6:** The statistics of the positioning times of the WKNN, LC-WKNN, and DFW-WKNN positioning algorithms in two experimental scenarios.

Scenarios	Method	Average	Min.	Max.	RMSE	95% Confidence Interval
Experimental Scenario 1 (*ms*)	WKNN	27.46	17	62	28.74	(24.64, 30.27)
	LC-WKNN	5.69	2	21	6.64	(4.55, 6.82)
	DFW-WKNN	4.37	1	13	4.93	(3.62, 5.13)
Experimental Scenario 2 (*ms*)	WKNN	94.67	17	157	95.71	(91.38, 97.96)
	LC-WKNN	18.19	6	43	19.06	(16.85, 19.52)
	DFW-WKNN	5.14	3	20	5.93	(4.45, 5.84)
